# A Type III CRISPR Ancillary Ribonuclease Degrades Its Cyclic Oligoadenylate Activator

**DOI:** 10.1016/j.jmb.2019.04.041

**Published:** 2019-07-12

**Authors:** Januka S. Athukoralage, Shirley Graham, Sabine Grüschow, Christophe Rouillon, Malcolm F. White

**Affiliations:** Biomedical Sciences Research Complex, School of Biology, University of St Andrews, North Haugh, St Andrews, Fife KY16 9ST, UK

**Keywords:** CARF, CRISPR-associated Rossman fold, HEPN, Higher Eukaryotes and Prokaryotes Nucleotide binding, cOA, cyclic oligoadenylate, cA_4_, cyclic tetra-adenylate, TTHB, *Thermus thermophilus* HB8, CRISPR, anti-viral signaling, cyclic oligoadenylate, ring nuclease, *Thermus thermophilus*

## Abstract

Cyclic oligoadenylate (cOA) secondary messengers are generated by type III CRISPR systems in response to viral infection. cOA allosterically activates the CRISPR ancillary ribonucleases Csx1/Csm6, which degrade RNA non-specifically using a HEPN (Higher Eukaryotes and Prokaryotes, Nucleotide binding) active site. This provides effective immunity but can also lead to growth arrest in infected cells, necessitating a means to deactivate the ribonuclease once viral infection has been cleared. In the crenarchaea, dedicated ring nucleases degrade cA_4_ (cOA consisting of 4 AMP units), but the equivalent enzyme has not been identified in bacteria. We demonstrate that, in *Thermus thermophilus* HB8*,* the uncharacterized protein TTHB144 is a cA_4_-activated HEPN ribonuclease that also degrades its activator. TTHB144 binds and degrades cA_4_ at an N-terminal CARF (CRISPR-associated Rossman fold) domain. The two activities can be separated by site-directed mutagenesis. TTHB144 is thus the first example of a self-limiting CRISPR ribonuclease.

## Highlights

• TTHB144 is a cyclic tetra-adenylate activated ribonuclease.
• TTHB144 degrades cyclic tetra-adenylate at its CARF domain.
• Self-limiting enzymes like TTHB144 may regulate anti-viral signaling in bacteria.

## Graphical Abstract

**Figure:**
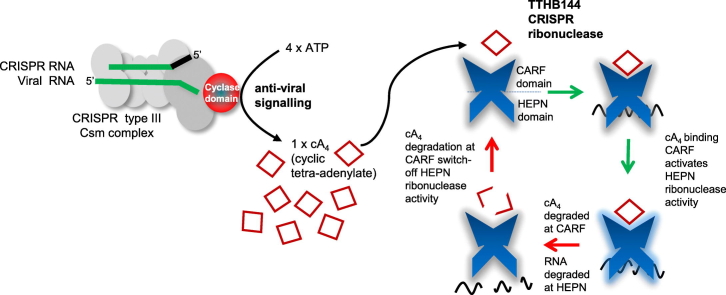

## Introduction

The CRISPR system provides prokaryotes with adaptive immunity against mobile genetic elements (reviewed in Refs. [1], [2], [3]). Type III (Csm/Cmr) CRISPR effector complexes harbor two nuclease activities for defense against mobile genetic elements: cleavage of foreign “target” RNA by the Cas7 subunit and degradation of single-stranded DNA by the HD nuclease domain (reviewed in Refs. [4], [5]). In addition, effector complexes produce cyclic oligoadenylate (cOA) anti-viral signaling molecules that activate CRISPR ancillary proteins to potentiate the immune response [6], [7]. On target RNA binding, the cyclase domain of the Cas10 subunit polymerises ATP into cOA, which consist of 3–6, 5′ to 3′-linked AMP subunits [6], [7], [8], [9]. cOA acts as an “alarm signal” within cells and strongly stimulates the activity of the CRISPR ancillary ribonucleases Csx1 and Csm6 [6], [7], [8]. Csx1/Csm6 family proteins consist of a CARF (CRISPR-associated Rossman fold) domain that binds cOA and a HEPN (Higher Eukaryotes and Prokaryotes Nucleotide binding) domain that possesses weak ribonuclease activity in the absence of cOA [10], [11]. Once stimulated by cOA, the non-specific RNA degradation activity of the Csm6 ribonuclease impacts both viral and cell growth [12]. Therefore, to recover from viral infection, cells require a mechanism for the removal of cOA. *Sulfolobus solfataricus (Sso)* encodes dedicated ring nucleases, which degrade the cyclic tetra-adenylate (cA_4_) activator and deactivate Csx1 [13]. Thus far, ring nucleases have only been identified in the crenarchaea, and, as highlighted by Mo and Marraffini [14], the enzyme(s) responsible for cOA degradation in bacteria remains unknown. The type III CRISPR system of the bacterium *Thermus thermophilus* HB8 (TTHB) has been investigated [15], [16], and its CRISPR ancillary ribonuclease TTHB152 was among the first shown to be activated by cA_4_ [6]. The type III CRISPR locus of *T. thermophilus* also encodes an uncharacterized CARF domain-containing protein, TTHB144, which was reported to be Csm6-like [17]. Here we report that TTHB144 is also a potent CRISPR ancillary HEPN ribonuclease activated by cA_4_. Furthermore, the enzyme degrades cA_4_ using its CARF domain. This enzyme therefore represents the first known example of a cOA dependent enzyme that degrades its own activator.

## Results and Discussion

**Fig. 1:**
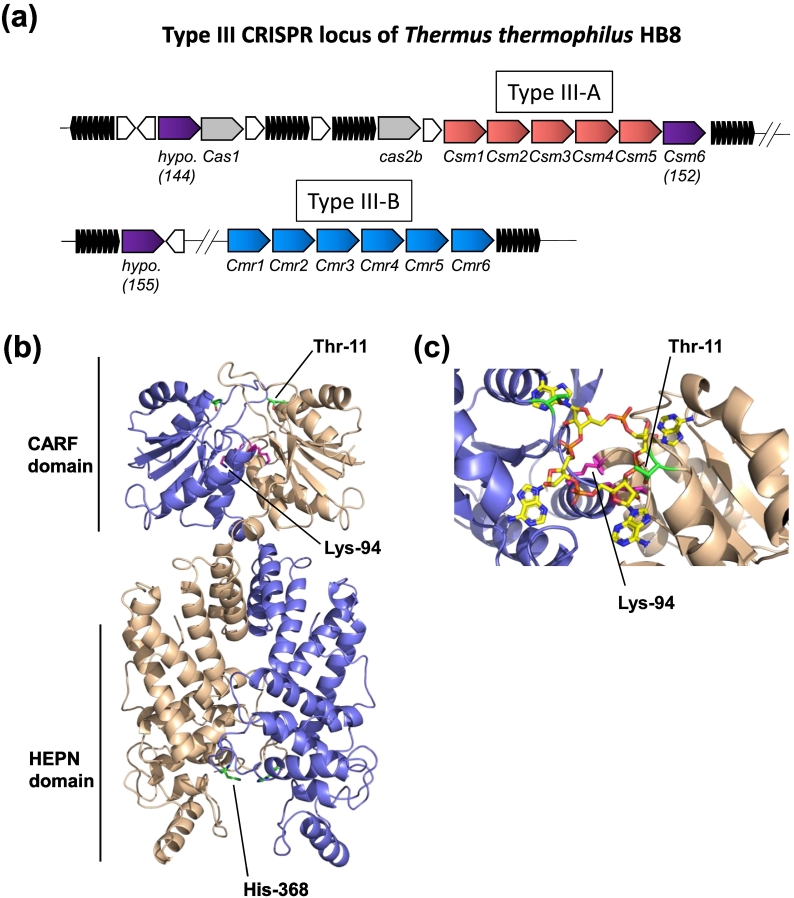
Type III CRISPR locus of *T. thermophilus* HB8 *(TTHB)* and model structure of TTHB144 with cA_4_ bound. (a) Gene neighborhood of *TTHB144* encoded on plasmid pTT27. Three genes encoding CARF domain-containing proteins (shown in purple) are present in the type III CRISPR locus of TTHB. TTHB152 is a Csm6 family protein, while TTHB144 and TTHB155 are hypothetical proteins of unknown function. (b) TTHB144 structure modeled using Phyre^2^. Each subunit of the predicted homodimer is shown by a different color (blue or cream). The highly conserved residues Thr-11, Lys-94 and His-368 are shown. (c) cA_4_ modeled into the CARF domain of TTHB144. Lys-94 is situated centrally beneath the cA_4_ molecule, and the side-chain of Thr-11 is suitably positioned to interact with cA_4_.

The *T. thermophilus* HB8 type III CRISPR locus encodes three CARF domain containing proteins, TTHB144, TTHB152 and TTHB155 (Fig. 1a). A x-ray crystal structure is available for TTHB152 (PDB: 5FSH) revealing a dimeric protein consisting of N-terminal CARF and C-terminal HEPN domains [10]. We modeled the structure of TTHB144 using the Phyre^2^ server [18], using TTHB152 as a template, and modeled cA_4_ into the electropositive pocket within the dimeric CARF domain (Fig. 1). Multiple sequence alignment identified highly conserved arginine and histidine residues within the HEPN domain characteristic of the Rx4-6H motif of HEPN nucleases [19]. Furthermore, we observed conserved lysine (K94) and threonine (T10/T11) residues within the ligand binding pocket of the CARF domain. By analogy with the ring nuclease Sso2081 [13], residues K94 and T10/T11 are suitably positioned to interact with the cA_4_ ligand. Consequently, we constructed a synthetic gene encoding TTHB144, expressed the protein in *Escherichia coli* using the plasmid pEV5hisTEV [20] and purified the recombinant protein using immobilised metal affinity and size exclusion chromatography, using methods described previously [21]. Site-directed protein variants were constructed and purified as for the wild-type enzyme.

**Fig. 2:**
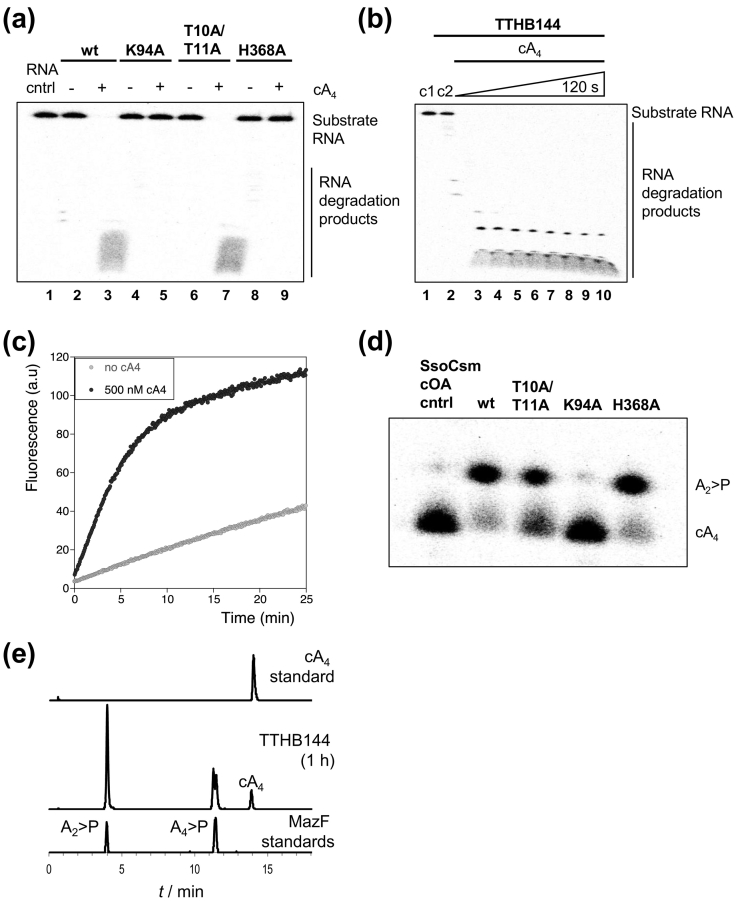
RNA degradation and cA_4_ cleavage occur in separate domains of TTHB144. (a) Phosphorimage of denaturing PAGE visualizing the degradation of 50 nM radiolabeled A1 RNA, as previously described [21], by TTHB144 (0.5 μM dimer), its CARF domain variants K94A and T10A/T11A and the HEPN domain variant H368A. The reaction was incubated at 70 °C for 60 min in pH 8.0 buffer containing 20 mM Tris–HCl, 150 mM NaCl, 1 mM EDTA and 3 units SUPERase•In™ inhibitor, before quenching by phenol–chloroform extraction. RNA was cleaved by wild-type (wt) protein and the T10A/T11A variant in the presence of 1 μM cA_4_, but not by the K94A or H368A variants. (b) Phosphorimage of denaturing PAGE visualizing degradation of 50 nM radiolabeled RNA by TTHB144 (1 μM dimer) when incubated with 20 μM cA_4_ at 70 °C. Control reactions incubating RNA with buffer (c1) or RNA with protein in the absence of cA_4_ (c2) are shown. All of the substrate RNA was degraded within 15 s (lane 3). (c) Plot of fluorescence emitted when RNaseAlert™ substrate (1.5 μM; Integrated DNA Technologies) was degraded by 125 nM dimer TTHB144 in the absence or presence of 500 nM cA_4_ at 50 °C. Fluorimetry was carried out in a Cary Eclipse Fluorescence Spectrophotometer (Agilent Technologies) with excitation and emission wavelengths set to 490 and 520 nm, respectively. (d) Phosphorimage of denaturing PAGE visualizing degradation of 400 nM radiolabeled cA_4_ generated using *Sso*Csm complex, as previously described [21], by TTHB144 (4 μM dimer) and variants at 70 °C for 120 min. cA_4_ was degraded to a slower migrating product. (e) High-resolution liquid chromatography mass spectrometry of cA_4_ produced using the *Sso*Csm complex and cleavage products generated on incubation with TTHB144 (40 μM dimer) at 70 °C. cA_4_ (~ 16 μM; top panel) was degraded to intermediate and product species (middle panel) with identical retention times to A_4_ > P and A_2_ > P, respectively (bottom panel). A_4_ > P and A_2_ > P standards were generated using the *E. coli* MazF toxin as previously described [8].

TTHB144 exhibited potent ribonuclease activity in the presence of cA_4_ and degraded RNA non-specifically (Fig. 2a). The H368A variant, targeting the HEPN active site, had no RNase activity, confirming that TTHB144 is a canonical HEPN ribonuclease. The T10A/T11A variant was still an active cA_4_-dependent ribonuclease, but the K94A variant was inactive, suggesting that cA_4_ no longer binds to activate the ribonuclease.

Subsequently, we assayed the rate of RNA degradation by TTHB144 under single-turnover conditions. TTHB144 fully degraded the RNA within 15 s (Fig. 2b), suggesting a lower limit of 5–10 min^−1^ for the catalytic rate constant. In addition, using the RNaseAlert™ fluorimetric assay system (Integrated DNA Technologies, USA) [6], we followed cA_4_-activated RNA cleavage by TTHB144 in a continuous assay (Fig. 2c). Consistent with observations made for other CRISPR ancillary ribonucleases such as *Enterococcus italicus* Csm6, *Streptococcus thermophilus* Csm6 and TTHB152 [10], this assay revealed weak TTHB144 ribonuclease activity, which was greatly enhanced by the addition of cA_4_.

To investigate whether TTHB144 degraded cA_4_, we incubated the wild-type protein with radiolabeled cA_4_ generated using the *S. solfataricus* type III-D Csm complex [21]. TTHB144 degraded cA_4_ to generate a slower migrating product on denaturing polyacrylamide gel electrophoresis (PAGE) (Fig. 2d), which we have previously identified as di-adenylate containing a 5′ hydroxyl moiety and a 2′,3′-cyclic phosphate (A_2_ > P) [13]. We verified this observation by high-resolution liquid chromatography–mass spectrometry, by comparison of cA_4_ degradation products with oligoadenylate standards generated using the *E. coli* MazF toxin, as described previously [21]. Similar to the *S. solfataricus* ring nucleases, TTHB144 degraded cA_4_ to yield an A_4_ > P intermediate and A_2_ > P product (Fig. 2e).

**Fig. 3:**
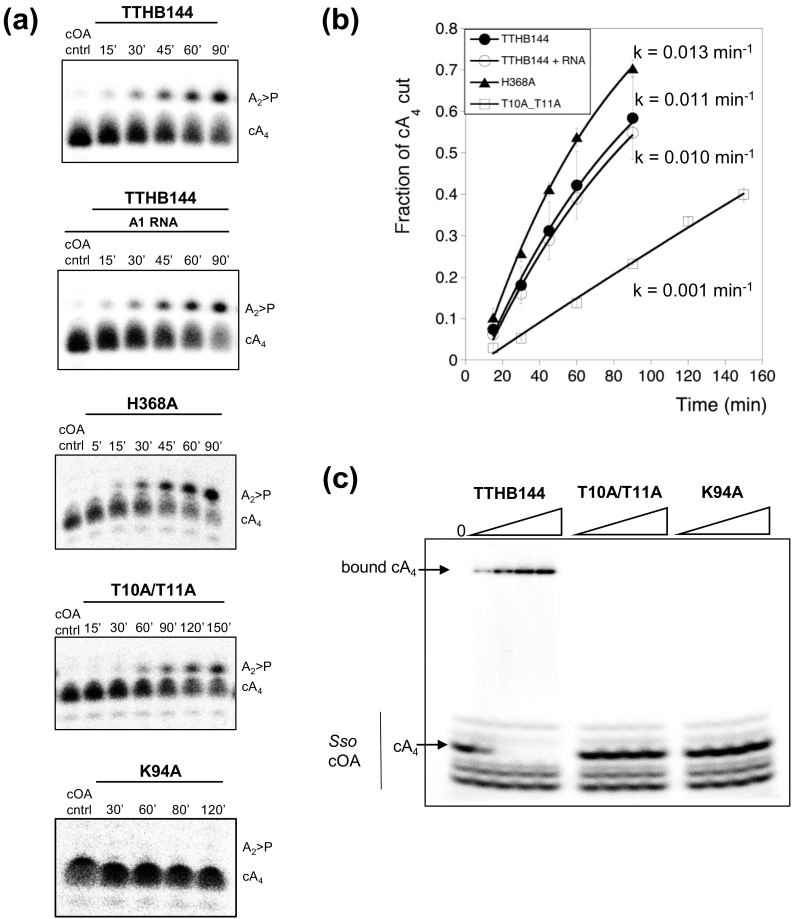
cA_4_ binding and cleavage by wild-type and variant TTHB144 enzymes. (a) Panels are phosphorimages of denaturing PAGE visualizing degradation of 200 nM radiolabeled cA_4_ by TTHB144 and variants (8 μM dimer) at 70 °C. cA_4_ is degraded to A_2_ > P. Time in minutes is indicated. Protein and radiolabeled cA_4_ were incubated in pH 8.0 buffer containing 20 mM Tris–HCl, 150 mM NaCl, 1 mM EDTA and 3 units SUPERase•In™ inhibitor, and reactions were quenched at the indicated time-points by phenol–chloroform extraction. A cA_4_ degradation assay was also carried out in the presence of 1 μM A1 RNA in order to evaluate the effect of RNA binding and cleavage at the HEPN active site on cA_4_ degradation at the CARF domain. (b) Plot of the fraction of cA_4_ cut *versus* time, generated by quantifying the densiometric signals from the phosphorimages depicted in panel a. All data points are the average of at least three technical replicates and are fitted to an exponential rise equation to derive the rate of cA_4_ degradation, as described previously [21]. Data points for TTHB144 are the average of six replicates encompassing two biological replicates with three technical replicates for each. Error bars show the standard deviation of the mean. (c) Electrophoretic mobility shift assay of radioactively-labeled cOA generated by the *S. solfataricus* Csm complex. cA_4_ is indicated; minor bands correspond to linear and cyclic byproducts of the reaction. cA_4_ (20 nM) was incubated with TTHB144 or variants T10A/T11A or K94A (0.1, 1, 10 or 20 μM protein dimer) in buffer containing 20 mM Tris–HCl (pH 7.5), 150 mM NaCl and 2 mM MgCl_2_ supplemented with 2 μM Ultrapure Bovine Serum Albumin (Invitrogen) for 10 min at 25 °C. A reaction volume equivalent of 20% (v/v) glycerol was then added prior to loading the samples on a 15% polyacrylamide, 1 × TBE gel. Electrophoresis was carried out at 25 °C and 200 V.

Subsequently, we evaluated cA_4_ degradation by TTHB144 CARF and HEPN domain variants. TTHB144 H368A, which has no ribonuclease activity, degraded cA_4_ similarly to wild-type protein, ruling out a role for the HEPN domain in cA_4_ degradation. However, cA_4_ degradation was abolished in the K94A variant and impaired in the T10A/T11A variant (Fig. 2d), suggesting a role for the CARF domain in this reaction. To confirm this hypothesis, we quantified the single-turnover rates of cA_4_ degradation by TTHB144 and its active site variants. The wild-type and H368A variant degraded cA_4_ at rates of 0.011 ± 0.004 and 0.013 ± 0.002 min^−1^, respectively (Fig. 3) allowing us to definitively rule out the HEPN domain as the site of cA_4_ degradation. The K94A variant was inactive, with no cA_4_ cleavage detectable over 2 h, while the T10A/T11A variant, which remains a cA_4_-activated HEPN ribonuclease (Fig. 2a), exhibited a ~ 12-fold decrease (*k* = 0.001 ± 0.002 min^−1^) in cA_4_ cleavage rate compared to the wild-type protein. The rate of RNA degradation (~ 5-10 min^−1^) thus appeared to exceed the rate of cA_4_ cleavage by approximately 3 orders of magnitude. Consequently, we investigated whether RNA binding at the HEPN domain stimulated cA_4_ degradation by the CARF domain by including unlabeled RNA in a cA_4_ degradation assay. TTHB144 degraded cA_4_ at a rate (0.010 ± 0.042 min^−1^) similar to cA_4_ degradation in the absence of RNA, suggesting that RNA binding at the HEPN domain does not affect cA_4_ degradation at the CARF domain.

To examine cA_4_ binding by the wild-type and variant enzymes, we carried out gel electrophoretic mobility shift assays with ^32^P-labeled cA_4_ (Fig. 3c). The wild-type protein bound the cA_4_ ligand at protein dimer concentrations as low as 100 nM, with 100% binding at 1 μM protein. In contrast, neither the T10A/T11A nor the K94A variants yielded detectable cA_4_ binding at protein dimer concentrations up to 20 μM. The highly conserved lysine residue K94 is clearly crucial for cA_4_ binding, and may also play a catalytic role during cA_4_ degradation. The T10 and T11 residues, which sit at the rim of the cA_4_ binding site, are also clearly important for cA_4_ binding, although the T10A/T11A variant does retain the ability to degrade (and therefore bind) cA_4_ at a reduced level. Hence, the TTHB144 cA_4_ binding and cleavage mechanism may be similar to that of the crenarchaeal ring nuclease Sso2081, where the equivalent residues, S11 and K106, have been shown to be important for cA_4_ binding and/or cleavage [13].

## Conclusions

TTHB144 is the first CARF family protein identified to harbor both cA_4_ degradation activity and ribonuclease activity. The single-turnover rate of cA_4_ degradation by TTHB144 at 70 °C is slow: comparable to that of the less active *S. solfataricus* dedicated ring nuclease, Sso1393 [13]. This slow rate of cA_4_ degradation may function as a built-in control mechanism to limit the extent of ribonuclease activity. Faster rates of cA_4_ degradation could disable this arm of type III CRISPR-mediated immunity. *Streptococcus epidermidis* Csm6 activity during type III immunity has been shown to cause cell growth arrest [12], and self-limiting enzymes may be crucial for cell recovery following clearance of invading genetic entities in bacteria that do not have dedicated ring nucleases. Therefore, the amalgamation of a HEPN ribonuclease and a ring nuclease into a single self-limiting enzyme may help decrease the toxicity associated with non-specific RNA cleavage in type III CRISPR systems.

## Acknowldgments

This work was funded by a grant from the Biotechnology and Biological Sciences Research Council (Grant REF BB/S000313/1 to M.F.W.).

**Author Contribution Statement:** J.S.A. carried out the investigation and analysis and wrote the original draft of the paper; S.Grü. and S.Gra. contributed to the investigation and analysis; M.F.W. conceptualized and supervised the work. All authors contributed to the review and editing.
